# Fire Behavior of Polyamide 12/Rubber Formulations Made by Laser Sintering

**DOI:** 10.3390/ma15051773

**Published:** 2022-02-26

**Authors:** Marcos Batistella, Monica Francesca Pucci, Arnaud Regazzi, José-Marie Lopez-Cuesta, Ouassila Kadri, David Bordeaux, Florence Ayme

**Affiliations:** 1 Polymers Composites and Hybrids (PCH), IMT Mines Ales, 30319 Ales, France; jose-marie.lopez-cuesta@mines-ales.fr; 2 LMGC, IMT Mines Ales, University Montpellier, CNRS, 30319 Ales, France; monica.pucci@mines-ales.fr (M.F.P.); arnaud.regazzi@mines-ales.fr (A.R.); 3 SDTech, 30100 Ales, France; ouassila.kadri@sd-tech.com (O.K.); david.bordeaux@sd-tech.com (D.B.); florence.ayme@sd-tech.com (F.A.)

**Keywords:** laser sintering, polyamide 12, rubber, fire behavior

## Abstract

In the present work, the processability and fire behavior of parts made by the laser sintering (LS) of polyamide 12/rubber powder blends is studied. In order to evaluate some of the interactions that could take place during LS, three acrylonitrile butadiene rubbers (NBRs) were used, which included two that had different acrylonitrile (AN) contents, and one that had carboxylated rubber. The results show that the flowability of the powders is strongly dependent on the rubber used. For the carboxylated rubber, a good flowability of the blend was observed, whereas the use of rubbers with different AN contents led to significant changes in the powder flowability, with a heterogeneous powder bed, and differences in the porosity as a function of the AN content. Furthermore, the addition of rubbers to polyamide 12 (PA12) entails an increase in the sintering window and, in particular, a change in the melting temperature of PA12 is noticed. Even though some changes in the crystallization and melting temperatures are observed, formulations containing 10 and 20 wt.% of rubbers could be processed using the same process parameters as PA12. Furthermore, the formulations containing carboxylated rubber show improved fire behavior, which is measured by a cone calorimeter, with reductions of about 45 and 65% in the peak of the heat release rate, compared to the PA12. Moreover, almost all of the samples evaluated in this study are classed as “Good” by the Flame Retardancy Index. This result can be partially explained by the formation of an amide linkage between the polyamide and NBR during processing, which could result in increases in the melt viscosities of these samples.

## 1. Introduction

Additive manufacturing (AM) is attracting attention in various industrial applications because of its freedom in terms of the part geometry, as well as the rapidity in the production of prototypes. AM technologies can be classified into seven categories as a function of the material state and the energy input. Among these, powder bed fusion (PBF) technologies are increasing its range of applications because of its greater freedom of part geometry, and the new materials that are currently available or under development [1]. There are several PBF technologies, which can be distinguished by the function of the heat source and the type of material. Among them, laser sintering (LS) uses a laser beam to selectively melt the polymer powder and build the parts. Moreover, because of the requirements of LS, only a few polymers and additives (e.g., flame retardants, glass beads) are currently available. In order to improve the range of additives that could be used in LS, various works have evaluated the addition of organomodified clays [2,3], multiwalled carbon nanotubes [4,5], and fire retardants [6,7]. The addition of these materials aims to improve the final properties of polyamide 12 (PA12), which is needed for most applications, such as in the aerospace industry. Moreover, the use of additives could have a negative effect on the processability of these blends because of the poorer flowability and, in some cases, the alteration of the laser/polymer interaction, which can strongly limit the sintering of the parts. Another way to improve the material properties is to use polymer blends. In this case, blending PA12 with a soft polymer could lead to an improvement in the mechanical, and possibly the fire properties, depending on the intrinsic fire behavior of the other polymer. In the LS research, few works have evaluated the use of polymer blends [8,9,10,11]. Salmoria et al. evaluated the processability of a PA12/polybutylene terephthalate (PBT) blend and found that the resulting mechanical properties depended on the PBT content [9]. Greiner et al. evaluated the processability of a miscible blend composed of polybutylene terephthalate and polycarbonate (PC) [8]. The results show that it was not possible to obtain dense parts with PBT. Moreover, the addition of PC led to a decrease in the porosity as a function of the PC content. These few results show that polymers should be selected carefully in order to obtain fully dense parts in LS that could be suitable for technical applications. Moreover, the use of some polymer blends may be interesting because of the chemical interactions that can occur between polymers during LS, which leads to some improvements in the part properties without previous processing, such as the extrusion in [8].

Apart from LS, various works in the literature have evaluated the blends between polyamides by classical methods (e.g., extrusion and injection molding) with polypropylene [12,13] and acrylonitrile butadiene rubbers [14,15], as well as with the use of compatibilizing agents, such as maleic anhydride-grafted polymers [16,17]. The use of polyamide/acrylonitrile butadiene rubber (NBR) blends has attracted some attention because of their ability to improve the toughness of polyamides and/or the oil resistance of NBRs [15]. However, the addition of cross-linkable polymers requires the use of curatives or compatibilizers in order to obtain the desired material properties. The effect of various cross-linking systems on butadiene acrylonitrile/polyamide 6 were evaluated by Mehrabzadeh and Delfan [18]. The authors found a phase inversion for a 60/40 (*w*/*w*) (NBR/PA6) composition, which led to a significant improvement in the tensile properties. It should be noted that the compositions of NBRs vary as a function of the acrylonitrile/butadiene ratio, which could have a significant influence on the crosslinking and, hence, on the mechanical properties of the blend. Cai et al. [15] evaluated the effect of the acrylonitrile (AN) content in PA6/NBR blends and found that the crystalline structure of the polyamides, as well as the melting and crystallization temperatures, were dependent on the AN content of the NBR. Mehrabzadeh and Burford evaluated the effect of the AN content (from 19 to 38 wt.% AN) in polyamide 11/butadiene-acrylonitrile blends [19], and they show a significant improvement in the impact strength of a 60/40 (*w*/*w*) (PA11/NBR) blend using an NBR with an intermediate AN content (corresponding to 33 wt.% of AN). Furthermore, in order to improve the compatibility between polyamides and NBRs, various modified NBRs have been evaluated, such as maleic-anhydride-grafted NBR [20,21], and carboxylated NBR [22,23]. Chowdhury et al. evaluated the influence of the carboxylation ratio on a PA/carboxylated NBR blend [22] and found that an increase in the number of carboxyl groups (from 1 to 7%) was able to improve the tensile strength and the elongation at break of all the evaluated blends. This improvement was attributed to a decrease in the interfacial tension, which can enhance the interfacial adhesion between the phases. The carboxylated functional groups of these NBRs can also react with the amine end groups of polyamides to form amide linkages, which are also likely to improve the properties of these blends.

Most of the works in the literature on polymer blends evaluate their influence on the mechanical properties, and a few works are concerned with the evaluation of the thermal properties and, particularly, the fire behavior of these blends, without the addition of flame retardants. Various routes are proposed in the literature to improve the fire behavior of polymers. The most studied and commercially available methodology is the use of flame retardants (FRs) as additives or, in order to achieve better performance through synergistic effects, the use of combinations of FRs. In the case of PA, various works deal with the use of phosphorous- and/or nitrogen-based flame retardants [24,25,26,27], and submicronic or nanofillers [28,29,30], as well as a combination of both [31]. However, the fire behavior of polymer blends with polyamides has received less attention in the literature. Most studies evaluating the thermal stability and fire behavior of polyamide blends deal with polypropylene/polyamide blends, with or without compatibilizers. Yet, in order to improve the flammability of polyamide blends, the same flame retardants are mostly evaluated as polymers alone [32,33]. To the best of our knowledge, only a few studies have evaluated blends of PA/NBR, but they only evaluate the mechanical properties. Hence, there is a lack of research on the influence of NBRs on the laser-sintering abilities and fire behavior of the resulting blends. Thus, the aim of this study is to evaluate the influence of AN/polyamide blends in terms of the processabilities, the thermal behaviors, and the fire reactions of parts made by LS.

## 2. Materials and Methods

PA12 (PA2200) powder, with a median diameter (D_50_) of 58 µm, was supplied by Electro Optical Systems (EOS, Krailling, Germany). Three NBRs in powder form were used: Nipol 1411 (D_50_ = 100 µm) and Zealloy 1422A (D_50_ = 100 µm), having different AN contents (38 and 33 wt.% respectively); and a pre-crosslinked carboxy-functionalized rubber (Duomod DP5045, D_50_ = 50 µm). All of the grades were provided by Zeon (Tokyo, Japan). Compositions with 10 and 20 wt.% of each NBR were prepared using a powder mixer station, P1, from EOS. Table 1 summarizes the different compositions prepared and the nomenclatures used through the text.

Three plates of 70 × 70 × 4 mm^3^ for the cone calorimeter tests, and six disks of 25-mm diameters and 1-mm thicknesses for the rheology measurements, were prepared using SnowWhite equipment from Sharebot. This printer uses a CO_2_ laser beam and has a build platform of 1.3 L. The process parameters were identical for every composition: a layer thickness of 0.1 mm; a hatching distance of 0.1 mm; a laser power of 3.5 W; and a laser scan speed of 2700 mm/s. These process parameters were obtained in previous studies as the optimal process parameters for sintering PA2200 using the SnowWhite printer [6]. In order to evaluate the effect of the processing on the rheological behavior, disks of 25-mm and 1-mm thicknesses were also prepared by compression at 80 °C, and a pressure of 20 bar.

The cone calorimeter tests were carried out using a fire testing technology (FTT) device with an irradiance of 50 kW/m^2^. The time to ignition (TTI), the peak of the rate release rate (pHRR), the total heat released (THR), the maximum of the average rate of the heat emission (MARHE), and the fire retardancy index (FRI) (Equation (1) [34]) were evaluated. For these tests, the samples (70 × 70 × 4 mm^3^) were placed in a sample holder with an aluminum foil. Smaller plates than the required ISO 5660-1 standard were printed because of some limitations of our equipment in terms of the specimen geometry. Nevertheless, according to Lindholm et al. [35], the downscaling of specimens is possible and, despite the fact that comparisons between larger specimens is not acceptable, comparisons between the reduced specimens with regard to the standard can be made. Three plates were tested for each formulation and the mean values are given in the tables.

(1) $$FRI=\frac{THR_{neat\;polymer}*\frac{pHRR_{neat\;polymer}}{TTI_{neat\;polymer}}}{THR_{composite}*\frac{pHRR_{composite}}{TTI_{composite}}}$$

The thermal stabilities of the polymers and blends were investigated using thermogravimetric analysis equipment (SETSYS Evolution from Setaram, Caluire-et-Cuire, France) under a nitrogen flow (40 mL/min), from 50 to 750 °C, at 10 °C/min. The differential scanning calorimetry measurements were performed using a Pyris Diamond DSC (Perkin-Elmer, Waltham, MA, United States). A temperature ramp of 10 °C/min was applied to investigate the fusion and crystallization phenomena that define the LS processing window. All of the tests were carried out on the powder mixtures. First, the heating and cooling cycles were analyzed. The crystallinities of the samples, $X_{c}$, were obtained in the second heating cycle, and were calculated using Equation (1), where $\Delta H_{m}^{{}^{\circ}}$ is the theoretical melting enthalpy for 100% crystalline polyamide 12 (i.e., 209.2 J/g [36]); ϕ is the weight fraction of the rubber in the blend; and $\Delta H_{m}$ is the melting enthalpy of the formulation:

(2) $$X_{c}=\frac{\Delta H_{m}}{\left(1-\phi \right)\Delta H_{m}^{{}^{\circ}}},$$

The SEM images of the cryofractured samples were obtained using an environmental scanning electron microscope (Quanta 200 FEG from the FEI Company, Hillsboro, OR, United States). The samples were coated with a carbon layer before observations. The rheological measurements were conducted with an Anton Paar rheometer, using a plate–plate geometry, at a temperature of 210 °C, a strain of 5%, and a frequency sweep from 10^−2^ to 10^2^ Hz.

The LS samples of PA12 containing carboxylated rubber were also prepared by means of an ultra-cryomicrotome (Leica EM UC7, Nussloch, Germany) in order to characterize their microstructures using atomic force microscopy (AFM, Oxford Instruments, Abingdom, England). The AFM setup was the MFP-3D Infinity by Asylum Research (Oxford Instruments, Abingdom, England). The tapping mode was used for these tests, with a TR400PB tip (resonant frequency of 32 kHz, and a spring constant of 0.09 N/m), at a scan rate of 1 Hz. Images of 5 × 5 µm^2^ were obtained.

## 3. Results

### 3.1. Influence of Additives on Sintering Window

The use of additives can significantly change the melting and crystallization temperatures of polyamides [6]. Generally, the addition of fillers can decrease the polymer chain mobility, which will lead to an increase in the melting temperature. Moreover, these additives could also act as nucleating agents with the increase in the melting temperature. In the case of rubber/polyamide blends, the polyamide molecular chains may have a strong interaction with the nitrile groups of NBR, which results in a decrease in the crystallization temperature and also limits the crystallization behavior of the polyamides [15]. In the case of PA12 used in LS, this behavior can lead to important changes in the sintering window, which is defined as the difference between the melting and crystallization temperatures. A significant decrease in the sintering window can lead to a build failure or to defects in the printed parts.

**Figure 1:**
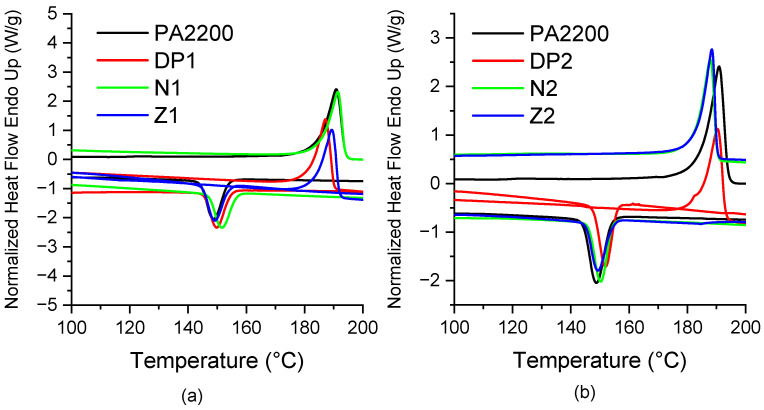
Thermograms of (**a**) formulations containing 10wt.% and (**b**) 20wt.% blends.

In order to evaluate the influence of the rubbers on the sintering window and the crystallinity of PA12, DSC measurements were carried out. The results are shown in Figure 1 and Table 2. PA2200 shows crystallization and melting onset temperatures of 153 and 179 °C, respectively, with a sintering window of 26 °C. This polyamide has a high melting enthalpy that is due to the high perfection of the crystals, which can be attributed to the way that they were manufactured (precipitation from a solution) [37]. Depending on the rubber type and content, the addition of rubbers leads to increases of 1–3 °C and 3–6 °C in the crystallization and melting onset temperatures of PA12, respectively. These differences lead to an increase of 1–4 °C in the sintering window, with higher values for the blends containing Zealloy 1422A rubber. The increase in the melting temperature is dependent on the amount of carboxylated rubber and the AN content. This suggests that the addition of rubbers may restrict the mobility of polymer chains. Furthermore, a decrease in the crystallization temperature of PA12 was observed for all the compositions containing 20 wt.% of rubber (cf. Table 2). Some studies in the literature show that the rubber molecules could reduce the hydrogen bond density of polyamides, which, in return, could limit the growth of spherulites, which results in a less ordered matrix [38]. The same conclusions were drawn for polyamides filled with mineral particles containing hydroxyl groups at their surface [39].

### 3.2. Morphologies, Porosities, and Thermal Stabilities of the LS Materials

**Figure 2:**
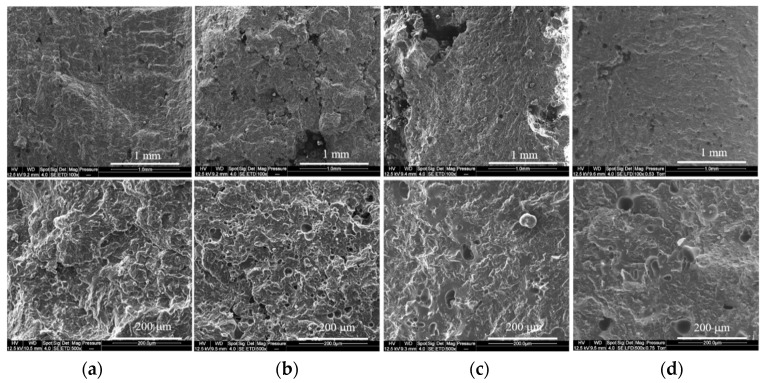
SEM images of LS printed parts at 100 and 500×: (**a**) PA2200; (**b**) DP2; (**c**) N2; and (**d**) Z2.

The use of additives or polymer blends in LS can lead to changes in the morphology of the printed parts because of the restriction of the polyamide chains mobility, the interface quality, and the polymer compatibility. One of the most common effects is an increase in the porosity, which would affect the mechanical properties and the fire behavior of the LS parts [6]. Thus, to evaluate the morphology of PA12/NBR blends, SEM observations of the cryofractured cross sections of blends containing 20 wt.% of each rubber were carried out and are presented in Figure 2.

From Figure 2a and Table 3, it can be noticed that the PA2200 sample has a low porosity content (about 4%), which is in agreement with the literature results [40,41]. The addition of carboxylated rubber (DP) results in a small increase in the porosity, with the production of pores of various sizes. Furthermore, the use of NBR with different AN contents (Figure 2c,d) leads to different behaviors. For the N2 formulation containing the highest AN content, it is possible to observe the formation of some pores with diameters in the order of 1 mm, which strongly limits its use. However, decreasing the AN content limits the pore size and amount, although the porosity is still significant, as is shown in Table 3. Nipol 1411 has a strong tendency to form agglomerates because of its higher AN content. These agglomerates tend to impart the flowability of the PA12/NBR blend, which leads to a heterogeneous powder bed, which partially explains the increased porosity of the formulations containing Nipol 1411. Furthermore, this NBR grade, in particular, could partially absorb the laser beam, which decreased the amount of energy transmitted to PA12, thereby increasing the porosity [42]. Yet, the reduced surface contact between PA12 and the NBR could also reduce the heat transfer, which limits the melting of the PA12 particles.

In order to evaluate the influence of the laser power on the formulations containing Nipol 1411, some tests were carried out with an increased laser power (results not shown). Increasing the laser power does not lead to a decrease in the porosity, but increased smoke production during the laser scan was observed, which could be related to polymer degradation. This behavior can indicate a strong interaction between the Nipol and the laser beam.

**Figure 3:**
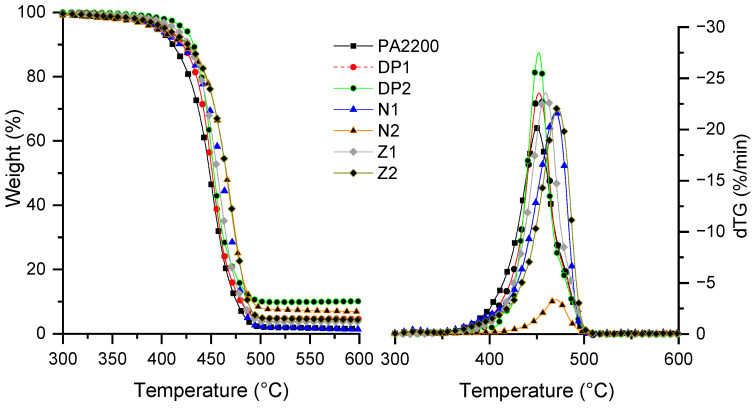
Mass loss curves of formulations under nitrogen at 10 °C/min.

The results of the thermogravimetric analyses of the PA12/NBR blends are given in Table 4, and the curves are shown in Figure 3. PA12 degrades in one step, with temperatures corresponding to 5 and 50 wt.% of losses (i.e., T_5%_ and T_50%_) of about 406 and 461 °C, respectively. All NBRs degrade in one step, as is shown in Table 4. The characteristic degradation temperatures of Nipol 1411 and Zealloy 1422 are very close to those of PA12, whereas a decrease in the T_5%_ and an increase in the T_50%_ are observed for Duomod DP5045. Furthermore, the residue at 750 °C is noticeable for all the NBRs, in contrast to neat PA12.

All polymer blends degrade in one step, with some variations in the characteristic temperatures. The addition of carboxylated NBR, at a 20 wt.%, leads to an increase of about 15 °C for the T_5%_, but a decrease of about 10 °C for the T_50%_ compared to PA12. The addition of Nipol 1411 leads to a decrease in the T_5%_, while no differences were observed for the T_20%_ and T_50%_. A decrease in the AN content (from N to Z formulations) leads to small changes in the characteristic temperatures as a function of the NBR ratio. Furthermore, a small increase in the TGA residue is observed at 750 °C, compared to the theoretical value, depending on the NBR and AN contents. The increases in the thermal stabilities that was observed for several formulations can be partially explained by some interactions (e.g., the hydrogen bonding, or the formation of amide linkages), which could result in a better interfacial adhesion between the components, which could limit the mass loss [43]. Furthermore, it should be noted that the crosslinked NBR segments might be able to delay the degradation of formulations containing higher amounts of NBR [43].

### 3.3. Fire Tests on LS Samples

**Figure 4:**
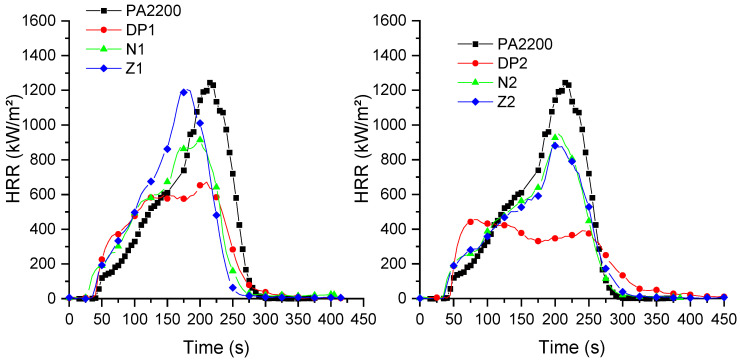
Heat release rates of compositions (irradiance: 50 kW/m^2^).

**Figure 5:**
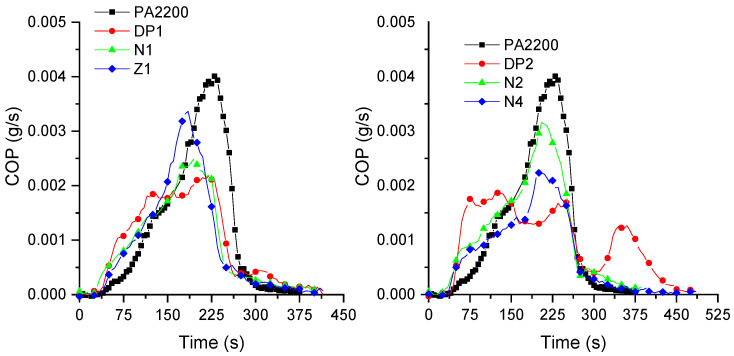
CO production of PA12 and PA12/NBR blends.

**Figure 6:**
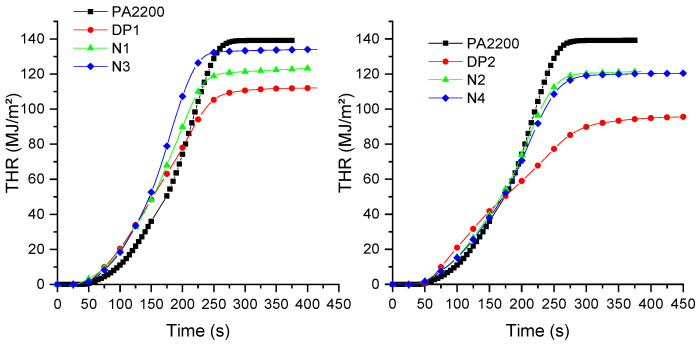
THR results of formulations.

Aliphatic polyamides are relatively highly flammable polymers. This strongly limits their use for technical applications. However, their fire behavior can be improved by the use of various flame retardants, particularly melamine cyanurate and phosphinates, possibly used in combination with mineral particles, as well as in a combination of both [31,44]. Polymer blends containing polyamides also need the use of flame retardants in order to improve their flammability [32]. However, it can be expected that low-flammable rubbers, which can form chemical bonds with polyamide end chains, could improve their fire behavior without the need for FR additives. In this case, an increase in the viscosity could decrease the mass transfer of the volatile products to the gas phase, which entails an improvement in the fire behavior. In order to evaluate the influence of the addition of rubbers in PA12, cone calorimeter tests were carried out on polymer blend samples [45,46,47], and the results are shown in Figure 4, Figure 5 and Figure 6 and Table 5. Three square specimens were tested, and the mean values are presented in Table 5.

Polyamide 12 burns very quickly, with a time to ignition (TTI) of 45 s, and a peak rate release rate (pHRR) of 1244 kW/m^2^. The addition of Nipol 1411 leads to a small decrease in the pHRR (of about 25%), but to an important decrease in the TTI for lower rubber contents (N1). Furthermore, decreasing the AN content (from N to Z samples) does not have a major impact on the TTI, but it increases the pHRR for the Z1 sample. The AN content has a major influence on the NBR flammability because of the presence of higher thermal stable chemical bonds. These bonds need higher amounts of energy in order to be broken [48], which explains the slightly better flammability of the N compared to the Z formulations. However, the higher amounts of AN also lead to the release of greater amounts of toxic gases, particularly carbon monoxide (CO) (Figure 5). It is interesting to note that, for formulations containing 10 wt.% of NBR, the increases in AN lead to a decrease in the CO production, whereas, for formulations containing 20 wt.%, it is the opposite. It is also interesting to note that the porosity seems to have a limited influence on the fire behavior. Regazzi et al. show that the porosity only has some influence on the TTI of the samples. This behavior was explained by a lower heat capacity that was due to the slightly lower mass of the samples [49]. Moreover, it can be assumed that the melting of the sample during the initial stage of heat exposure will lead to a collapse of the pore structure.

The addition of carboxylated NBR leads to interesting decreases in the pHRR, of about 54% for DP1, and 65% for DP2. For this last composition, a plateau is observed for the HRR values, which indicates a thick charring behavior [50]. A decrease in the CO production can also be observed compared to the neat polyamide and the formulations without carboxylated NBR. Moreover, it is possible to observe two CO peaks at about 250 s and 350 s, without significant mass losses at these times. The conversion of CO to CO_2_ is a complex reaction, and it is relatively slow compared to other reactions. Furthermore, the increase in the smoke toxicity was shown in the literature for some NBRs as a function of the AN ratio, which was related to the formation of CO, HCN, and NO_2_ [48].

Only small differences were observed for the EHC and the MARHE, except for the MARHE value corresponding to the DP2 composition. Moreover, the residue is lower than expected, indicating that there was some further degradation of the rubbers during the cone calorimeter test.

Vahabi et al. defined a universal dimensionless index to describe the fire behavior of samples made by different polymers and flame retardant systems [34]. As a function of the FRI values, the different compositions can be classified as “Poor”, “Good”, and “Excellent”. Samples having an FRI lower than 1 are classified as “Poor”, those with an FRI between 1 and 10 are classified as “Good”, and for FRIs higher than 10, the sample is classified as “Excellent”. As can be observed from Table 5, all the samples can be classified as having “Good” fire performances, except the formulation containing the rubber with the lower AN content. This classification also shows that the addition of rubbers having different AN contents at 10%wt negatively impacts the flame retardancy of these formulations.

The presence of the functional groups can also partially explain the differences with regard to the fire behavior of the formulations. It is shown in the literature that an increase in the viscosity might be able to reduce the pHRR. This behavior was associated with a reduction in the volatile gas transfer from the condensed to the gas phase, with an increase in the flaming time [51,52]. In order to evaluate the changes that could be promoted using rubbers, viscosity measurements were carried out.

### 3.4. Rheological Measurements

The literature results show that blending polyamides with NBRs could lead to different effects. In particular, if some chemical interactions between polymers occur because of the presence of reactive groups or the use of compatibilizers, an improvement in the mechanical properties can be achieved or, in some cases, a phase inversion can be obtained as a function of the ratio between the polymers, and/or as a function of the crosslinking of the rubber. These changes in the microstructure and the influence of specific chemical interactions may also lead to important changes in the viscoelastic behavior of the blends and, in some cases, to changes in their fire behavior.

**Figure 7:**
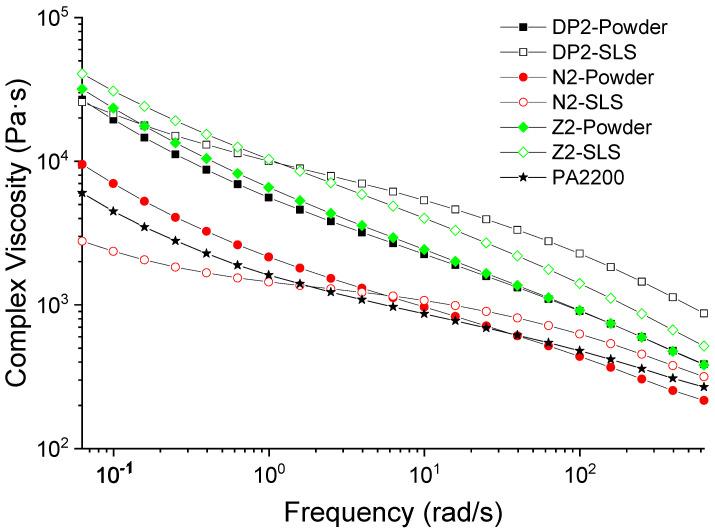
Complex viscosities of PA12 and PA12/NBR blends at 210 °C as a function of angular velocity. Unfilled symbols: LS samples; filled symbols: thermocompressed samples.

In order to evaluate the influence of the processing, samples containing 20 wt.% of NBR were made by LS and by thermocompression. Figure 7 shows their complex viscosities. The viscosities of all the samples decreased with the frequency, which indicates a shear-thinning behavior. The addition of the NBR containing the higher amount of AN (N2) led to a small increase in the complex viscosity compared to PA2200. Furthermore, a decrease in the AN content (Z2), and the use of the carboxylated NBR (DP2), in particular, led to an additional increase. In LS, no shear is applied to the molten polymer, and the powder particles, once melted, coalesce. As a result, the phenomenon observed in extrusion (phase inversion, changes in the morphology of the dispersed phase), which could impact the measured viscosity, are not observed in LS. Nevertheless, if specific physicochemical interactions between the polymers occur during LS processing, the particular viscoelastic behavior of the blend could be observed. Interestingly, by comparing the viscosities of the samples made by LS and thermocompression (Figure 7), an increase in the viscosities of the LS samples is observed. Consequently, these results suggest that some physicochemical interactions between the constituents could occur during LS.

### 3.5. FTIR Measurements

**Figure 8:**
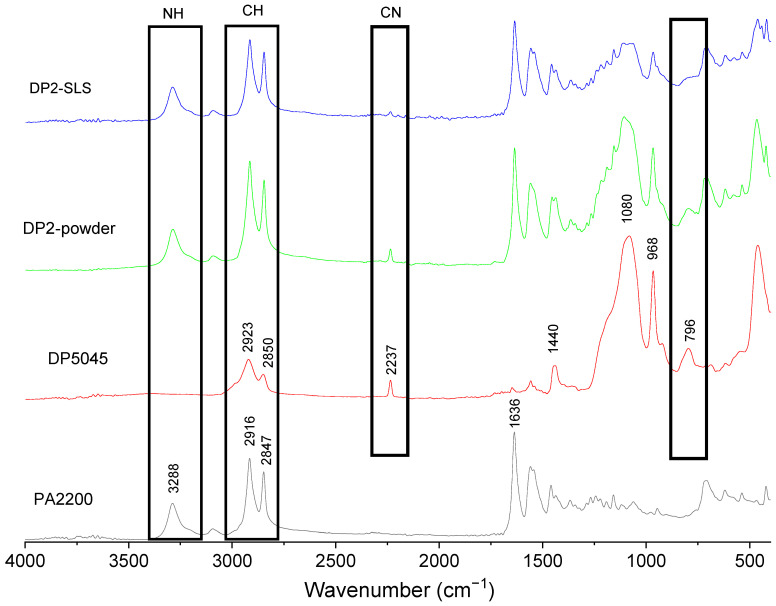
FTIR results of formulations containing 20 wt.% of carboxylated NBR.

In order to understand the possible interactions between the polyamides and the carboxylated NBR, FTIR-ATR measurements were conducted on powders and on LS samples, and the results are shown in Figure 8. PA12 shows the typical vibration bands at 3288 cm^−1^, which can be assigned to the stretching vibration of NH. The bands at 2916 and 2847 cm^−1^ corresponds to CH_2_ stretching, and the peak at 1636 cm^−1^ can be attributed to C=O stretching. The spectra of the carboxylated NBR shows the characteristic peaks of CH stretching (1923 and 2850 cm^−1^), and =CH out-of-plane bending (968 cm^−1^). The band at 2237 cm^−1^ can be attributed to the stretching vibration of the C≡N groups of AN. The band at 1440 cm^−1^ can be assigned to the OH bend in the carboxylic acid of the NBR. Finally, the bands at 1080 cm^−1^ and 796 cm^−1^ can be attributed to the NH wag of the secondary amines [53].

As was expected, the spectra of the powder (DP2 formulation) seem to be a combination of those of PA12 and NBR. However, for the LS sample, some differences can be noticed. First, a decrease in the –CN peak amplitude is observed, which indicates some degradation of the –CN groups, or the formation of amide linkages with the carboxyl end groups of polyamide chains. Furthermore, it is possible to observe an important reduction in the band corresponding to the secondary amines (796 cm^−1^), which indicates the formation of amide linkages.

In the present study, the melting and coalescence of polyamide powder is highly dependent on the NBR content. Increasing the amount of NBR to 40 wt.% leads to a very porous structure, in which PA12 particles are still visible, even with an increase in the energy density (cf. Figure S1 in Supplementary Materials). Furthermore, the transmittance of the powder affects the energy distribution with, in some cases, a higher thermal gradient along the vertical (Z) axis. This could be more significant for formulations containing higher amounts of carboxylated rubber, which would explain the increased porosity for the samples containing 40 wt.% of the carboxylated rubber.

**Figure 9:**
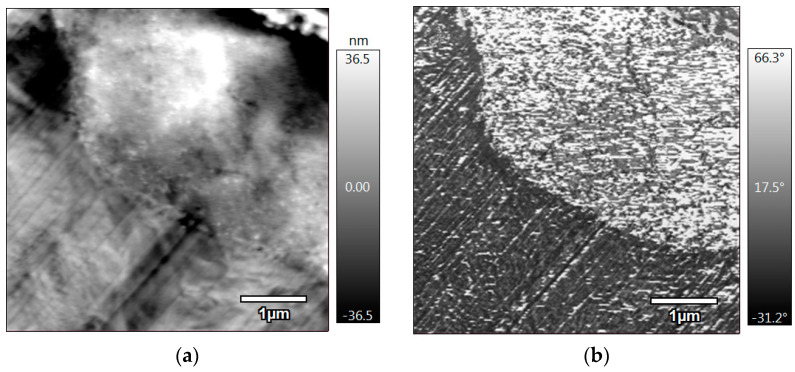
(**a**) AFM topography, and (**b**) phase images of DP2 formulation.

The AFM topographic and phase images of the formulation containing 20 wt.% of carboxylated rubber (DP2) are shown in Figure 9. The topography shows that the obtained surface was flat and smooth (the range of the roughness was inferior to 100 nm), with the black regions indicating pores due to the LS process, which is coherent with the SEM images. However, the phase image shows good adhesion between PA12 (the black zone corresponding to the stiffest material) and the carboxylated NBR (the white zone corresponding to the softest material), which confirms that some type of chemical interaction occurred.

## 4. Conclusions

The use of polymer blends in LS can help to increase the range of applications of LS with improved properties, such as the fire behavior. Moreover, it is important to carefully select the polymer blend in order to be able to print dense parts. In this case, the addition of polymers that were able to interact chemically with polyamides led to some improvements in terms of the fire behavior.

In the present work, PA12/NBR blends were prepared and successfully printed using LS. The addition of these NBRs positively affects the sintering window of PA12, with an increase from 26 to 30 °C as a function of the NBR composition, which allows for the building of parts with the same parameters as PA12. Furthermore, blending PA12 with NBRs does not significantly change the thermal stability of PA12, as measured by the TGA. It can be noticed that the flammabilities of the blends are very different as a function of the rubber type. For the rubbers having different contents of AN, slightly better fire behavior was observed, with a small decrease in the pHRR as a function of the loading, compared to neat PA12. However, the addition of the carboxylated rubber leads to a greater decrease in the pHRR. Yet, almost all the samples were classified as having a “Good” fire performance by the FRI index. These differences are ascribed to the chemical interactions between the carboxyl groups of the NBR, and the amine end chain groups of PA12, which leads to the formation of amide linkages. These interactions result in an increased viscosity of PA12, which partially explains the observed behavior. 

## Figures and Tables

**Table 1:** Compositions of PA12/NBR blends.

Sample	PA2200 (wt.%)	Duomod DP5045 (wt.%)	Nipol 1411 (wt.%)	Zealloy 1422A (wt.%)
PA	100	0	0	0
DP1	90	10	0	0
DP2	80	20	0	0
N1	90	0	10	0
N2	80	0	20	0
Z1	90	0	0	10
Z2	80	0	0	20

**Table 2:** Crystallization (*T_ic_*) and melting (*T_im_*) onset temperatures, and crystallization (*T_c_*) and melting (*T_m_*) peak temperatures of PA12 and PA12/NBR blends.

Sample	$T_{ic}$ (°C)	$T_{im}$ (°C)	ΔT (°C)	$T_{c}$ (°C)	$T_{m}$ (°C)	*X_c_* (%)
PA2200	153	179	26	149	190	21
DP1	155	182	27	145	187	20
DP2	156	184	28	152	190	16
N1	156	185	29	152	191	19
N2	154	182	28	150	188	15
Z1	154	184	30	149	189	19
Z2	153	183	30	149	188	12

**Table 3:** Internal porosity values of LS samples.

Sample	Porosity (%)
PA	4
DP1	5
DP2	6
N1	8
N2	12
Z1	4
Z2	6

**Table 4:** Characteristics of the degradation of PA, NBR, and PA12/NBR blends.

Sample	T_5%_ (°C)	T_20%_ (°C)	T_50%_ (°C)	Experimental Residue at 750 °C (%)	Theoretical Residue at 750 °C (%)
PA2200	406	438	461	-	-
Duomod DP5045	384	436	477	43	-
Nipol 1411	404	438	465	33	-
Zealloy 1422	402	438	465	25	-
DP1	404	435	451	3.9	4.2
DP2	421	440	453	10.6	8.4
N1	395	439	461	0.5	3.2
N2	392	444	465	4.4	6.4
Z1	413	439	456	3.7	2.5
Z2	403	445	465	3.7	5

**Table 5:** Cone calorimeter results of PA12 and PA12/NBR blends.

Sample	TTI (s)	pHRR (kW/m²)	EHC (kJ/g)	THR (MJ/m²)	MARHE (kW/m²)	Residue (%)	FRI	COP (g/s)
PA2200	45 ± 8	1244 ± 150	32 ± 1	139 ± 4	518 ± 20	-		0.004 ± 0.0003
DP1	35 ± 2	705 ± 54	33 ± 0.5	116 ± 7	434 ± 14	-	2.8	0.0022 ± 0.0002
DP2	40 ± 2	442 ± 25	31 ± 0.5	110 ± 10	313 ± 17	-	3.1	0.0015 ± 0.0002
N1	34 ± 5	1005 ± 78	32 ± 1	132 ± 9	491 ± 7	-	1	0.0028 ± 0.0005
N2	39 ± 2	920 ± 80	32 ± 1	118 ± 15	449 ± 74	1	1.4	0.0025 ± 0.0002
Z1	38 ± 8	1276 ± 141	33 ± 1	132 ± 9	557 ± 45	-	0.9	0.0022 ± 0.0006
Z2	41 ± 2	1013 ± 115	33 ± 1	124 ± 4	513 ± 90	-	1.2	0.0023 ± 0.0002

## Data Availability

Not applicable.

## Supplementary Materials

The following supporting information can be downloaded at: https://www.mdpi.com/article/10.3390/ma15051773/s1, Figure S1: SEM image of a composition containing 40 wt.% of carboxylated rubber.
