# Supplementary material for: Fire Behavior of Polyamide 12/Rubber Formulations Made by Laser Sintering
Source: Materials (Basel). 2022 Feb 26;15(5):1773. doi: 10.3390/ma15051773 (PMC8911470; doi:10.3390/ma15051773)
Supplement: Supplementary file 1 [file materials-15-01773-s001.zip › materials-1555174-supplementary.pdf]

Supplementary Materials

Fire Behavior of Polyamide 12/Rubber Formulations Made by Laser Sintering

Marcos Batistella [1,*], Monica Francesca Pucci [2], Arnaud Regazzi [2], José-Marie Lopez-Cuesta [1], Ouassila Kadri [3], David Bordeaux [3] and Florence Ayme [3]

[1] Polymers Composites and Hybrids (PCH), IMT Mines Ales, 30319 Ales, France; jose-marie.lopez-cuesta@mines-ales.fr
[2] LMGC, IMT Mines Ales, University Montpellier, CNRS, 30319 Ales, France; monica.pucci@mines-ales.fr (M.F.P.); arnaud.regazzi@mines-ales.fr (A.R.)
[3] SDTech, 30100 Ales, France; ouassila.kadri@sd-tech.com (O.K.); david.bordeaux@sd-tech.com (D.B.); florence.ayme@sd-tech.com (F.A.)
* Correspondence: marcos.batistella@mines-ales.fr

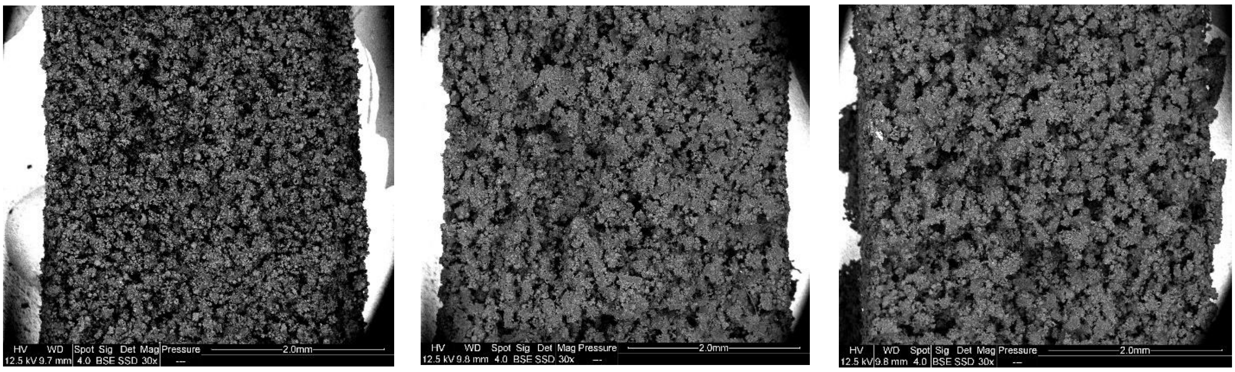

(**a**) (**b**) (**c**)

Figure S1. SEM images of LS printed parts at 30 x of samples containing 40 %wt. of carboxylated rubber using different laser power : (**a**) 3.5W, (**b**) 4W and (**c**) 4.5W.
